# The impact of medical errors on healthcare professionals: a cross-sectional propensity score-matched comparison of second victim syndrome between Japan and the United States

**DOI:** 10.1186/s13054-026-06212-5

**Published:** 2026-07-31

**Authors:** Yudai Takatani, Taro Minami, Genta Kato, Gerardo P. Carino, Shigeru Ohtsuru

**Affiliations:** 1 Department of Primary Care and Emergency Medicine, Kyoto University Graduate School of Medicine, Kyoto, Japan; 2 Division of Pulmonary, Critical Care, and Sleep Medicine, The Warren Alpert Medical School of Brown University, Providence, RI USA; 3 Division of Pulmonary, Critical Care, and Sleep Medicine, Kent Hospital, Care New England, Warwick, RI USA; 4 Solutions Center for Health Insurancec Claims/Department of Hospital Capacity Management, Kyoto University Hospital, Kyoto, Japan

**Keywords:** Cross-cultural comparison, Medical errors, Patient safety, Psychological distress, Second victim syndrome

## Abstract

**Background:**

Medical errors cause significant psychological distress among healthcare professionals, known as “second victim syndrome” (SVS). Although SVS is universal, it is likely modulated by organizational and cultural contexts. Therefore, we aimed to address the following question: Do cultural and legal differences between Japan and the United States (US) influence emotional distress and coping behaviors among healthcare professionals following medical errors?

**Methods:**

This cross-sectional, web-based survey included healthcare professionals at 14 Japanese hospitals, and used validated clinical scenarios identical to a prior US study. To enable rigorous comparison, 1:1 propensity score matching was performed based on specialty, profession, and years of clinical experience.

**Results:**

A total of 542 Japanese healthcare professionals responded (response rate: 40.0%). After matching, 185 pairs (*n* = 370) were analyzed. A “psychological burden paradox” emerged: despite reporting lower actual involvement in severe medical errors than US counterparts (11.9% vs. 73.0%, *P* < .001), Japanese professionals experienced more intense distress. Following a severe error, Japanese professionals reported a higher prevalence of guilt (94.6% vs. 61.6%), anxiety (84.9% vs. 55.7%), and fear of litigation (76.2% vs. 42.2%) (all *P* < .001). Furthermore, Japanese respondents were more than three times as likely to consider leaving their profession (30.8% vs. 8.6%, *P* < .001). Despite perceiving greater institutional support (83.8% vs. 70.6%, *P* = .012), Japanese providers were more likely to “just move on” (34.1% vs. 11.4%, *P* < .001) and less likely to discuss the event with colleagues (48.6% vs. 61.1%, *P* = .027).

**Conclusion:**

Significant cultural disparities exist in SVS symptoms and coping behaviors. Japanese healthcare professionals experience disproportionately intense psychological distress despite less frequent error exposure, likely driven by cultural norms, emphasizing shame and a legal framework that criminalizes medical errors, leading to a “culture of silence.” Support systems in Japan must be tailored to address these unique structural and legal barriers.

**Graphical Abstract:**

**Supplementary Information:**

The online version contains supplementary material available at https://doi.org/10.1186/s13054-026-06212-5.

**Figure:**
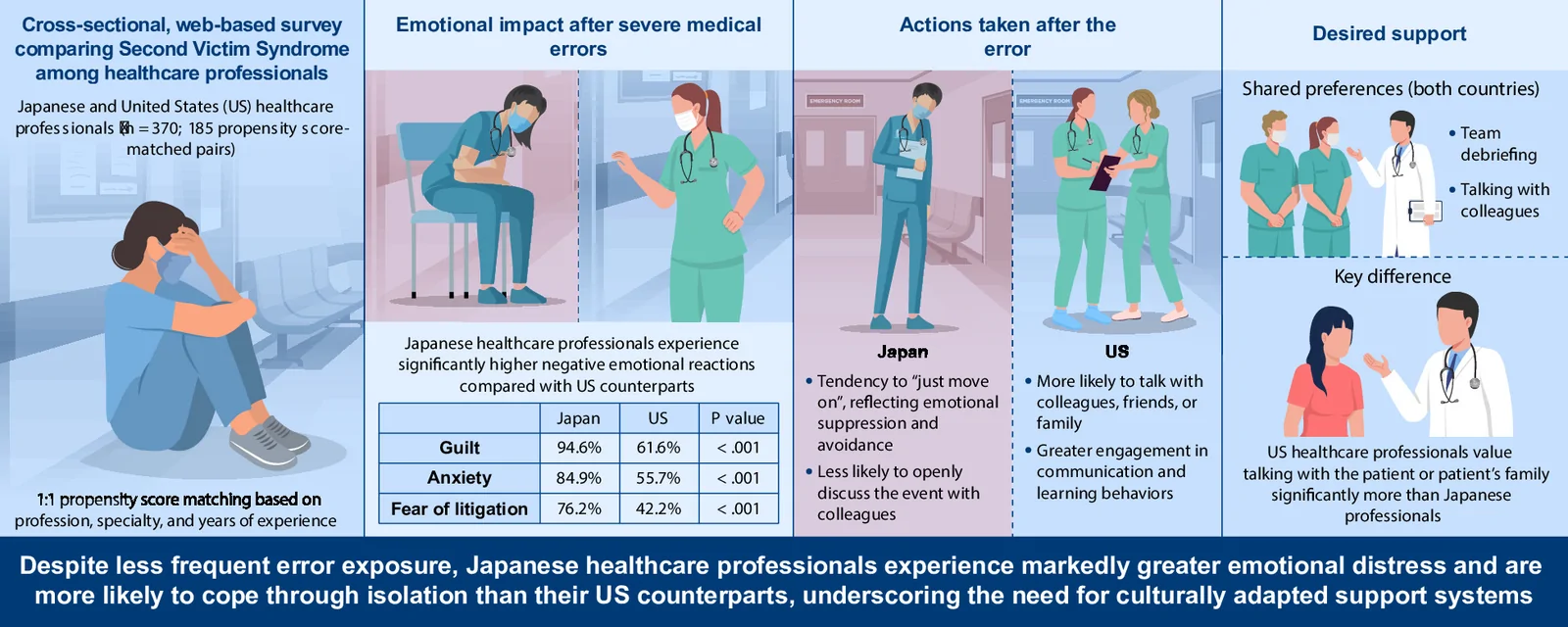

## Background

Medical errors represent a significant threat to patient safety, particularly in the high-stakes environment of critical care [1–4]. While their impact on patients is well documented, the emotional toll on healthcare professionals—known as “second victim syndrome” (SVS)—has only recently gained attention [5]. Healthcare providers in critical care settings are especially vulnerable given the complexity and severity of the cases they manage [6–9]. A previous study in the United Sates (US) revealed that critical care providers experience significant distress and fear of disciplinary action following medical errors [10]. However, SVS is not solely an individual psychological reaction, and is likely influenced by the surrounding organizational and cultural context [11, 12]. Although epidemiological data indicate that medical error and adverse event rates in acute care settings are broadly comparable across developed countries [8, 9], institutional safety cultures differ considerably. For instance, prior cross-cultural research suggests that Japanese healthcare professionals perceive their organizations as more punitive and less supportive of error disclosure than their US counterparts [13]. Nevertheless, few studies have quantitatively compared how these divergent organizational and cultural contexts shape SVS—including both emotional reactions and subsequent coping behaviors—across different cultural settings.

Japan represents a compelling comparator to the US. Despite similarly advanced healthcare systems, Japan is characterized by distinct sociocultural norms related to shame, collective responsibility, and an implicit expectation of “medical infallibility” [14, 15]. Furthermore, Japan’s legal environment permits criminal investigation of medical errors under Article 21 of the Medical Practitioners’ Act [16], in contrast to the primary civil liability framework in the US. Understanding how these cultural and legal backgrounds contexts influence both emotional responses and subsequent behaviors among healthcare providers is essential for developing effective, culturally-sensitive support systems.

We hypothesized that the Japanese cultural and legal context significantly intensifies the severity of SVS and promotes more avoidant coping behaviors. To test this hypothesis, we conducted a nationwide survey of Japanese healthcare professionals using the same validated scenarios employed in the prior US study and performed a propensity score-matched analysis to rigorously compare the impact of medical errors between the two countries.

## Methods

### Study design and ethical considerations

This study used a cross-sectional, anonymous, web-based survey design. Data collection was conducted in two phases due to the COVID-19 pandemic: initially at Kyoto University Hospital, a large-scale tertiary care academic medical center, from December 2019 to November 2020, followed by data collection at 13 affiliated teaching and community hospitals between March and July 2022. At each facility, departmental representatives responsible for the care of critically ill patients distributed the survey link to eligible participants. Data were collected using Google Forms. The study protocol was approved by the Ethics Committee of the relevant institution (Approval No. R2170). This study was reported in accordance with the STROBE (Strengthening the Reporting of Observational Studies in Epidemiology) guidelines.

### Participants and data sources

To assess the impact of errors in a cross-cultural context, data from this study were compared with historical control data from a US study conducted by Kaur et al. [10]. Among the US respondents, four participants with missing information on key background variables required for matching (i.e. specialty, profession, and years of experience) were excluded. Consequently, the final unmatched dataset included 897 US respondents and 542 Japanese respondents.

### Questionnaire design

The survey instrument was based on the questionnaire used in the previous US study [10] with modifications to ensure cultural and clinical relevance. It assessed: (1) demographic characteristics; (2) expectations of criticism or disciplinary action across three scenarios (Scenario 1: Procedural Error, Scenario 2: Diagnostic Error, and Scenario 3: Medication Error); (3) emotional reactions, including a question regarding prior involvement in a medical error resulting in severe morbidity or death; (4) institutional support; (5) behavioral responses; and (6) desired support tools. A brief summary of these scenarios and the evaluated outcomes is provided in Table 1. The full questionnaire is provided in the Supplementary Material.

**Table 1:** Summary of clinical scenarios and outcomes in the survey

Scenario type	Brief description	Outcomes evaluated (severity)
**Scenario 1**: **procedural error**	An error occurring during a clinical procedure (e.g., arterial injury or pneumothorax during central line placement)	1. No complications (Minor) 2. Extended hospital stay (Moderate) 3. Patient death (Fatal)
**Scenario 2**: **diagnostic error**	A diagnostic error resulting in inappropriate treatment (e.g., administering heparin for aortic dissection misdiagnosed as acute coronary syndrome)	1. No harm (Minor) 2. Shock requiring blood transfusion (Moderate) 3. Patient death (Fatal)
**Scenario 3**: **medication error**	Administration of a contraindicated medication (e.g., administration of penicillin to a patient with a documented allergy)	1. No adverse effect (Minor) 2. Anaphylactic shock (Moderate) 3. Patient death (Fatal)

For each outcome, respondents were asked to assess the likelihood of criticism from colleagues and formal disciplinary action. In Scenario 3: Medication Error, the clinical scenario was modified from that used in the original US study to improve clarity regarding the contraindication

The original English questionnaire was translated into Japanese. Two major modifications were made. First, the categories for “level of training” and “primary area of focus” were adjusted to align with the Japanese medical specialty system. Second, the clinical scenario for Scenario 3: Medication Error was simplified: whereas the US study described the administration of ceftriaxone to a patient with penicillin allergy, the present study described the administration of penicillin to a patient with a penicillin allergy to improve clarity. Additionally, the scenario/question assessing prior involvement in medical errors was presented as a direct, positively phrased question in the Japanese survey, rather than the negatively phrased option within a “check-all-that-apply” list used in the US survey.

### Outcome measures

**Fig. 1:**
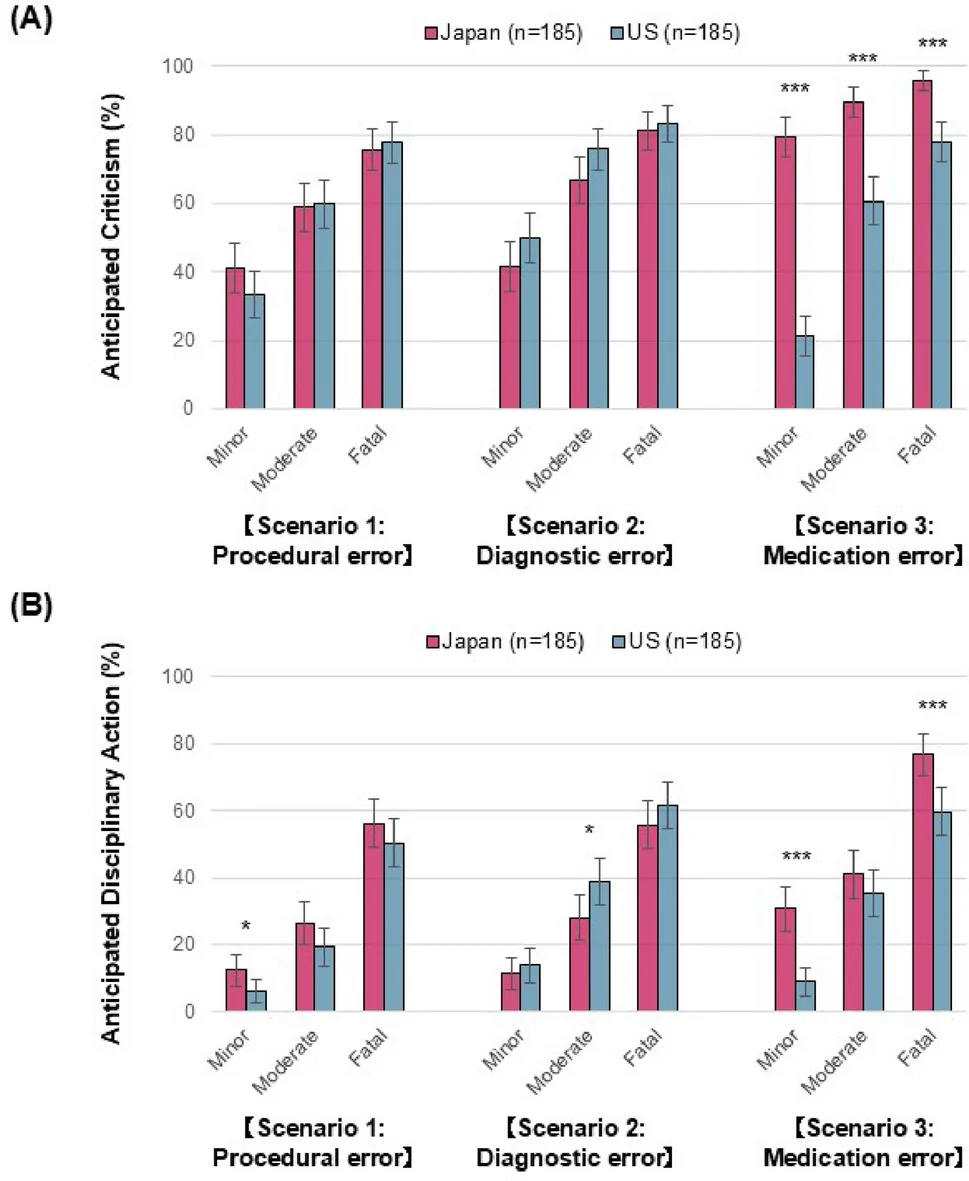
Comparison of Anticipated Responses to Medical Errors Between Japan and the United States. (**A**) Anticipated criticism from colleagues. (**B**) Anticipated formal disciplinary action. Percentages represent the proportion of healthcare professionals who responded with “almost certainly,” “very likely,” or “likely” on a 5-point Likert scale across three clinical scenarios (Scenario 1: Procedural Error; Scenario 2: Diagnostic Error; Scenario 3: Medication Error) and three levels of severity. Comparison was performed between propensity score-matched cohorts of Japanese (red bars) and US (blue bars) professionals (*n* = 185 per group). Error bars indicate 95% confidence intervals. Statistical significance for ordinal differences between the matched pairs was determined using the Wilcoxon signed-rank test. In Panel A, Japanese professionals displayed a significantly higher anticipation of criticism across all severity levels in Scenario 3: Medication Error. In Panel B, while Japanese professionals generally anticipated more formal disciplinary action, US professionals were significantly more likely to expect such actions for moderate outcomes in Scenario 2: Diagnostic Error. *P < .05, ***P < .001 for Japan vs. the United States

**Fig. 2:**
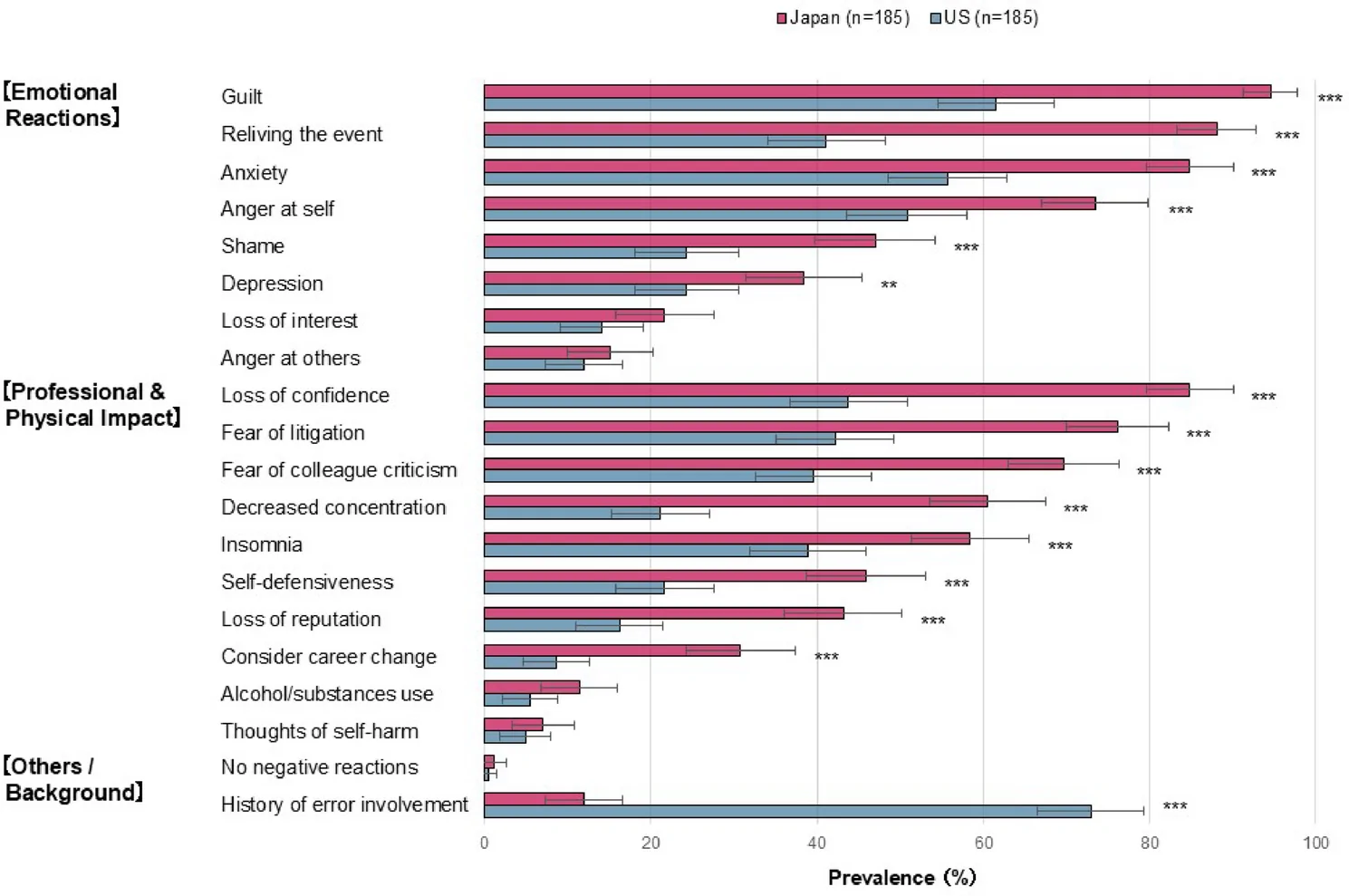
Comparison of negative emotional and behavioral reactions following medical errors. Percentages of Japanese (red bars) and US (blue bars) healthcare professionals who reported specific psychological and professional consequences are shown (*n* = 185 per group), categorized into psychological, professional/physical, and other impacts. Data represent the proportion of respondents who selected each item in a multiple-choice, “check-all-that-apply” format. Japanese professionals reported significantly higher rates of negative reactions across nearly all domains, including guilt, reliving the event, and loss of professional confidence. *P < .05, **P < .01, ***P < .001 for Japan vs. the United States

**Fig. 3:**
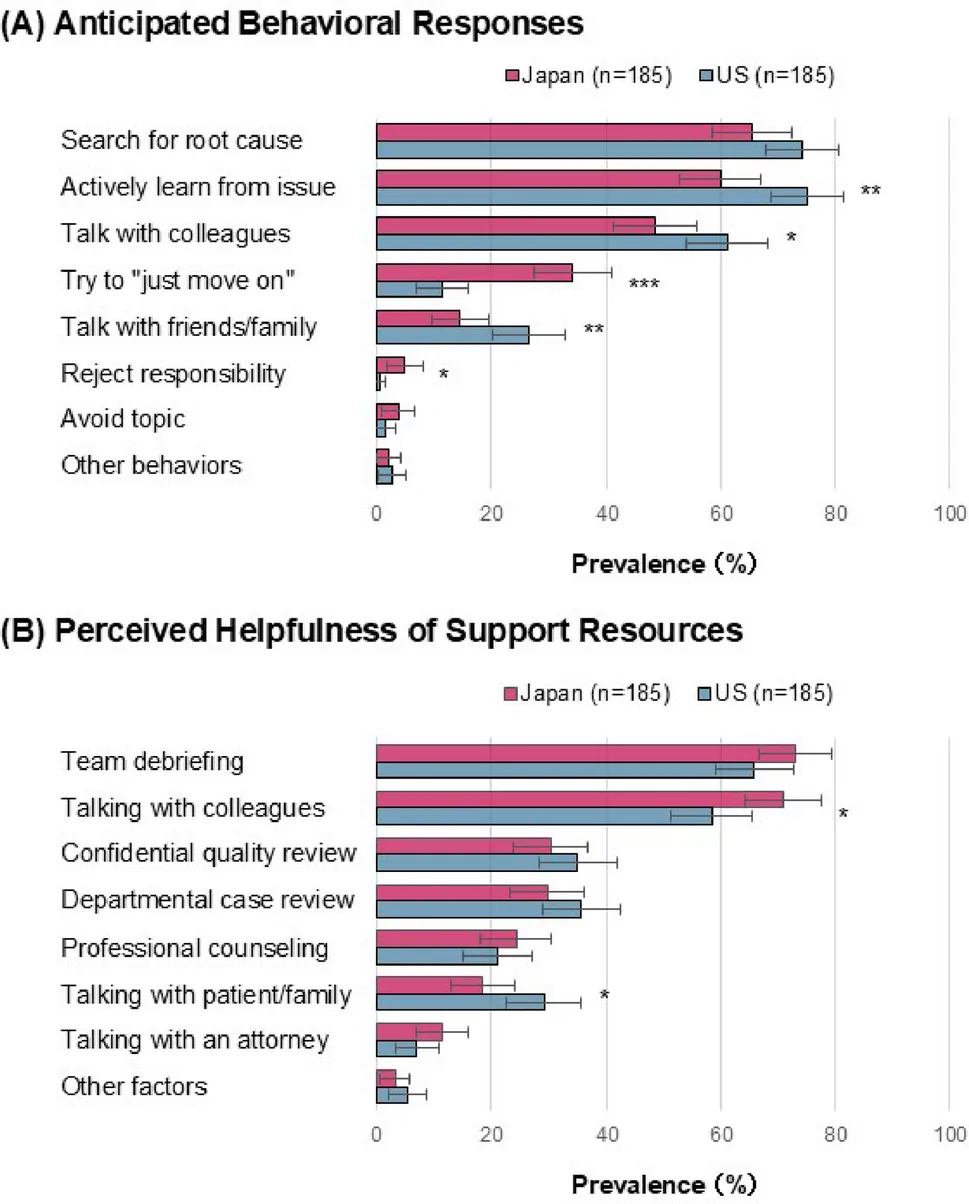
Behavioral Responses and Perceived Helpful Factors Following Severe Medical Events. (**A**) Anticipated behavioral responses to severe medical events. Percentages represent the proportion of healthcare professionals who reported specific behavioral responses they would expect to take following a hypothetical severe medical event. Items are sorted in descending order based on the response rates in Japan. (**B**) Perceived helpfulness of support resources and factors. Percentages represent the proportion of respondents who identified each resource or action as "helpful" if they were to experience such an event. Comparison was performed between propensity score-matched cohorts of Japanese (red bars) and US (blue bars) professionals (n = 185 per group). Error bars indicate 95% confidence intervals. Statistical significance was determined using the McNemar test for paired proportions. In Panel A, Japanese professionals were significantly more likely to try to "just move on" compared to US professionals. In Panel B, while both groups identified team debriefing and talking with colleagues as the most helpful factors, US professionals were significantly more likely to value talking with the patient or their family. *P < .05, **P < .01, ***P < .001 for Japan vs. the United States

Consistent with the methodology of the original US study [10], we evaluated multiple dimensions of the impact of medical errors. The primary outcome was the difference in the prevalence of negative emotional reactions between Japanese and US healthcare professionals. Secondary outcomes included behavioral responses, institutional support, fear of repercussions (criticism and disciplinary action), and the prevalence of a self-reported history of error involvement. The assessment formats varied by outcome: fear of repercussions (Fig. 1) was measured using a 5-point Likert scale ranging from “almost certainly” to “almost certainly not,” which was dichotomized into “anticipated” (including “almost certainly,” “very likely,” and “likely”) and “not anticipated” (including “not likely” and “almost certainly not”) for analysis. Negative emotional reactions (Fig. 2) and behavioral responses (Fig. 3) were evaluated using a multiple-choice, “check all that apply” format. Institutional support was assessed using a binary (Yes/No) question.

### Statistical analysis

Item-level missing data were infrequent and handled using available-case analysis for each comparison. Multiple imputation was not performed because the primary objective was item-specific group comparisons and the proportion of missing data was minimal. To reduce confounding due to baseline differences, propensity-score matching was performed. Propensity scores were estimated using a logistic regression model including specialty, profession, and years of experience. Japanese and US respondents were matched in a 1:1 ratio using the nearest-neighbor method without replacement, with a caliper width of 0.05. Covariate balance was assessed using absolute standardized differences (ASD), with values < 0.1 indicating negligible imbalance. Comparisons between matched cohorts were conducted by treating the data as paired samples. Categorical variables were compared using McNemar’s test, and continuous or ordinal variables were compared using the Wilcoxon signed-rank test. All statistical analyses were performed using IBM SPSS Statistics for Windows, Version 29.0 (IBM Corp., Armonk, NY). A two-sided P value < 0.05 was considered statistically significant.

## Results

### Participant characteristics and matching

A total of 542 Japanese healthcare professionals completed the survey (response rate: 40.0%). Before matching, significant differences were observed between the Japanese and US cohorts with respect to profession and years of experience (Table 2, Unmatched Cohort). After propensity score matching to adjust for baseline differences, 185 pairs (*n* = 370) of Japanese and US healthcare professionals were included in the final analysis. The matched cohorts were well-balanced regarding profession (Physician: 44.9% vs. 48.6%), specialty (Emergency/Critical Care: 14.1% vs. 16.2%), and years of experience (11 + years: 55.7% vs. 59.5%). ASD for all covariates were < 0.1, indicating negligible imbalance (Table 2, Matched Cohort).

**Table 2:** Baseline characteristics of healthcare professionals in Japan and the United States before and after propensity score matching

Variable	Unmatched cohort			Matched cohort		
	Japan (*n* = 542)	US (*n* = 897)		Japan (*n* = 185)	US (*n* = 185)	
	n (%)	n (%)	ASD	n (%)	n(%)	ASD
**Profession**			1.157			0.092
Physician	124 (22.9)	654 (72.9)		83 (44.9)	90 (48.6)	
Nurse	322 (59.4)	142 (15.8)		68 (36.8)	67 (36.2)	
Pharmacist	14 (2.6)	83 (9.3)		14 (7.6)	13 (7.0)	
Other	82 (15.1)	18 (2.0)		20 (10.8)	15 (8.1)	
**Specialty**			1.584			0.06
Emergency/critical care	337 (62.2)	34 (3.8)		26 (14.1)	30 (16.2)	
Anesthesiology	4 (0.7)	98 (10.9)		4 (2.2)	4 (2.2)	
Internal Medicine/surgery/stroke	135 (24.9)	487 (54.3)		109 (58.9)	106 (57.3)	
Pediatrics	9 (1.7)	190 (21.2)		9 (4.9)	8 (4.3)	
Other	57 (10.5)	88 (9.8)		37 (20.0)	37 (20.0)	
**Years of experience**			0.21			0.077
In training	7 (1.3)	44 (4.9)		5 (2.7)	5 (2.7)	
0–5 years	146 (26.9)	233 (26.0)		52 (28.1)	48 (25.9)	
6–10 years	105 (19.4)	173 (19.3)		25 (13.5)	22 (11.9)	
≥11 years	284 (52.4)	447 (49.8)		103 (55.7)	110 (59.5)	

Data are presented as n (%) unless otherwise indicated. Abbreviations: ASD, absolute standardized difference. The US dataset was obtained from a historical control study by Kaur et al. [10]. Propensity score matching was performed in a 1:1 ratio using the nearest-neighbor method with a caliper width of 0.05. Covariates included specialty, profession, and years of experience. An ASD < 0.1 indicates negligible imbalance between the two groups

### Criticism and disciplinary action

The anticipation of disciplinary action varied by error severity and country (Fig. 1). In the minor procedural error scenario (Scenario 1: Procedural Error), Japanese professionals were significantly more likely than US professionals to anticipate formal disciplinary action (12.4% vs. 6.1%, *P* = .036). Similarly, in the fatal medication error scenario (Scenario 3: Medication Error), Japanese professionals were significantly more likely to anticipate criticism from colleagues (95.7% vs. 77.9%, *P* < .001) and to expect formal disciplinary action (76.8% vs. 59.8%, *P* < .001) compared with their US counterparts. In contrast, there was no significant difference in the anticipation of disciplinary action for minor or fatal outcomes in the diagnostic error scenario (Scenario 2: Diagnostic Error). Notably, for Scenario 2: Diagnostic Error resulting in moderate disability, US professionals were significantly more likely than Japanese professionals to anticipate formal disciplinary action (38.7% vs. 28.1%, *P* = .032).

### Self-reported involvement and emotional impact

Regarding the history of medical error involvement, a striking disparity was observed: only 11.9% of Japanese professionals reported ever being involved in a medical error resulting in severe morbidity or death, compared with 73.0% of their US counterparts (*P* < .001). Following a hypothetical severe error, Japanese professionals reported a substantially higher prevalence of negative emotional reactions (Fig. 2). Specifically, they were more likely to experience feelings of guilt (94.6% vs. 61.6%, *P* < .001), anxiety (84.9% vs. 55.7%, *P* < .001), and fear of litigation (76.2% vs. 42.2%, *P* < .001) compared with the US group. Notably, 30.8% of Japanese professionals indicated they would consider leaving their profession or job following such an event, compared with 8.6% of US professionals (*P* < .001).

### Coping behaviors and helpful factors

Regarding institutional culture, a significantly higher proportion of Japanese respondents perceived their hospital administration as supportive compared with US respondents (83.8% vs. 70.6%, *P* = .012). However, behavioral responses differed significantly between groups (Fig. 3A). Japanese professionals were more likely to report that they would “try to just move on” (34.1% vs. 11.4%, *P* < .001) and were less likely to “actively learn from the issue” (60.0% vs. 75.1%, *P* = .005) or discuss the event with colleagues (48.6% vs. 61.1%, *P* = .027) than US professionals. In terms of resources perceived as helpful (Fig. 3B), a greater proportion of Japanese professionals perceived talking with colleagues as beneficial compared with US professionals (70.8% vs. 58.4%, *P* = .016). Conversely, US professionals were more likely to value talking with the patient or their family (29.2% vs. 18.4%, *P* = .025). Both groups rated team debriefing as the most helpful resource (73.0% vs. 65.9%, *P* = .171).

## Discussion

This study represents the first propensity score-matched comparison of the emotional and behavioral impact of medical errors between Japanese and US healthcare professionals. Our findings reveal a striking disparity: despite adjusting for professional background, Japanese professionals exhibited significantly higher levels of anxiety, guilt, and fear of litigation following a hypothetical severe error (Fig. 2). Notably, even for the minor outcome in Scenario 1: Procedural Error, Japanese respondents were more likely to anticipate formal disciplinary action (Fig. 1). Furthermore, a behavioral paradox emerged: although Japanese professionals perceived their institutions as supportive, they were substantially less likely to utilize this support, instead opting to “minimize the event and just move on” (Fig. 3A) and expressing a threefold higher intention to leave their profession compared with their US counterparts (Fig. 2).

### Independent vs. interdependent self

The contrast in emotional responses, specifically that Japanese providers predominantly feel “guilt” and “anxiety” while US providers experience “anger at self” [10], suggests a fundamental cultural difference in how responsibility is processed. In the US, where individual autonomy and an independent self-concept are emphasized [17], errors may be viewed as personal performance failures, triggering self-directed anger. In contrast, Japanese culture is characterized by an interdependent self-concept and is described as a “shame culture,” with errors perceived as disrupting group harmony (*wa*) or betraying the patient’s trust. This cultural tendency likely promotes internalization of failure as guilt and shame rather than externalization or constructive processing [17, 18]. This intense fear of shame may lead to subconscious avoidance of the recognition or acknowledgment of errors [19], creating a “culture of silence” that hinders system improvement [20, 21]. Crucially, this cultural pressure does not imply that clinicians internally deny the occurrence of an error; rather, they appear to bear the resulting guilt in isolation. This unexpressed emotional distress may manifest as a substantial psychological burden that remains largely hidden yet ultimately contributes to attrition from clinical practice.

### The communication paradox

A notable “communication paradox” emerged regarding peer support. Although Japanese professionals were significantly more likely to perceive “talking with colleagues” as a helpful resource compared with their US counterparts (Fig. 3B) they were paradoxically less likely to engage in such discussions following an error (Fig. 3A). This discrepancy suggests that while Japanese providers instinctively seek peer validation to alleviate guilt, the fear of burdening the team (*meiwaku*) acts as a powerful deterrent. In a culture where maintaining relational harmony is paramount, the act of seeking support may be viewed as an additional burden on colleagues, leading to emotional suppression and isolation.

### Legal barriers and fear

For moderate outcomes in Scenario 2: Diagnostic Error (Fig. 1B), US professionals anticipated significantly more frequent disciplinary action than Japanese professionals, suggesting qualitative differences in how medical accountability is perceived. In the US, the “Just Culture” framework and established patient safety systems may foster a more consistent threshold for accountability, where even non-fatal errors in diagnostic reasoning are viewed as subject to institutional review. In contrast, the fear of punishment among Japanese professionals appears to be more “context-specific” and “outcome-dependent.” This disparity is likely rooted in Japan’s unique medico-legal environment, particularly the influence of Article 21 of the Medical Practitioners’ Act [16]. This statute requires physicians to report any “abnormal death” to the police within 24 h, potentially bypassing institutional review processes and triggering criminal investigations. The ambiguity surrounding the definition of an “abnormal death” has contributed to criminal investigations involving allegations of professional negligence. Although the Medical Accident Investigation System (MAIS) was established in 2015 to promote organizational learning and a “no-blame” culture, recent evidence suggests that MAIS reports—originally intended for learning and recurrence prevention—are frequently used as evidence in medical malpractice litigation and may contribute to determinations of clinical negligence [22]. Ohira et al. argue that this possibility may discourage reporting and undermine the intended function of the system. They further noted that, unlike in the United States, Japan lacks statutory protections that prevent the use of such reports in legal proceedings [22]. This distinction highlights a fundamental difference between the two systems. In the US, critical-incident reporting is intended primarily to improve patient safety, and criminal prosecution is generally reserved for cases involving intentional harm or gross negligence [16]. In Japan, however, criminal investigation under allegations of professional negligence remains a more prominent concern [16]. Consequently, even when hospitals adopt “no-blame” approaches or conduct root-cause analyses, clinicians may perceive that the findings of internal reviews could later be used in legal proceedings [16, 22]. The profound gap in self-reported error involvement observed in our study likely reflects this structural fear, which elevates the “threshold for recognition” for medical errors in Japan. In an environment where errors are inextricably linked to criminality, Japanese providers may unconsciously avoid the “error” label except in the most undeniable and catastrophic cases. Consequently, the extreme sensitivity of Japanese providers to fatal outcomes and clear procedural lapses (Fig. 1) is likely amplified by this structural fear of criminal liability, which overshadows institutional support. Even if a hospital offers peer support [23], the lack of legal protection for open disclosure inhibits true psychological safety. Such emotional suppression, driven by a lack of psychological safety, is strongly associated with burnout and decreased patient safety [24]. Conversely, when the outcome is not fatal and the error involves diagnostic uncertainty (Fig. 1), Japanese professionals may perceive it more as an unavoidable clinical challenge rather than a punishable offense, explaining their lower anticipation of formal sanctions for moderate errors compared with their US counterparts.

### Workforce implications: the “30% risk”

The most alarming finding of our study is that nearly one-third of Japanese professionals would consider leaving their profession or job following a severe error (Fig. 2). This rate is more than three times that of the US cohort and highlights a critical workforce vulnerability. SVS is not merely an individual well-being issue, but a systemic threat. A “psychological burden paradox” emerged: despite far fewer Japanese professionals reporting prior involvement in severe medical errors, their emotional distress was significantly greater. Given that the objective incidence of clinical errors in Japanese intensive care units appears comparable to international benchmarks [8, 9], this contrast suggests that the low rate of self-reported error involvement observed in Japanese studies does not reflect safer clinical environments but rather a profound fear of blame and underreporting, consistent with Japan’s more punitive safety culture ratings [13]. In practice, this structural pressure to withhold error disclosure may render the associated psychological trauma largely invisible. By utilizing standardized hypothetical scenarios, however, our study minimized real-world confounding factors and enabled assessment of the psychological impact of medical errors under comparable conditions. Consequently, the internalized guilt and emotional distress that clinicians may otherwise experience in silence became evident through the markedly high anticipated rates of leaving the profession observed in our cohort. In Japan, failing to address these structural barriers could accelerate the collapse of regional medical systems, especially when combined with the lack of psychological safety and subsequent burnout [24]. 

### Implications for support systems

Our findings suggest that simply importing US-style support systems, such as standard peer support teams [23], is insufficient for Japan. While support availability is perceived as high, utilization is blocked by cultural stoicism and legal concerns. Future support systems in Japan must be culturally tailored and non-punitive. First, legal reform or clarification regarding the boundaries between medical error and criminal liability is essential to ensure true psychological safety [16]. Second, Japanese professionals struggle to initiate informal peer support despite their high perceived need (Fig. 3B). Formal and structured institutional systems, such as mandatory team debriefings, may be more effective because they shift the burden of “asking for help” from the individual to the system. Crucially, these interventions must address the profound feelings of shame and guilt [25, 26], characteristic of Japanese culture, rather than focusing solely on general stress management. Such targeted support is warranted to prevent the loss of skilled professionals and mitigate the “30% risk” of workforce attrition.

### Strengths and limitations

Strengths of this study include the use of standardized scenarios and propensity score matching to enable rigorous cross-national comparison. However, several limitations must be noted. First, unmeasured confounders, such as gender, hospital characteristics (e.g., teaching vs. community hospitals), and cultural nuances, may exist despite matching. Additionally, although years in practice were balanced between the two cohorts through propensity score matching to isolate macro-level cultural effects, we did not perform stratified analyses by career stage. Previous research suggests that less experienced healthcare professionals face greater distress and fear of litigation following medical errors because of their developing clinical confidence [11, 12]. Whether this vulnerability among early-career professionals is further amplified by Japan’s hierarchical culture or medico-legal environment remains an important area for future research. Second, while the response rate was 40.0%, selection bias may still exist; providers with strong negative experiences may have avoided the surveys. Third, the study relied on hypothetical scenarios. However, previous research suggests a correlation with actual responses [27]. Finally, the marked difference in self-reported error involvement must be interpreted with caution. The discrepancy in phrasing—positive in the Japanese version versus negative in the US version—and the item’s position at the end of the list may have introduced response or recall bias. Furthermore, cultural modifications to the questionnaire may have influenced participants’ interpretations.

## Conclusion

This study demonstrates that cultural and legal contexts profoundly shape the emotional and behavioral responses of healthcare professionals to medical errors. While Japanese healthcare professionals exhibit higher emotional distress and a more intense fear of punishment in specific clinical contexts, particularly those involving fatal outcomes or clear procedural lapses, US providers show a more consistent threshold for accountability. This “culture of silence” in Japan is likely reinforced by both cultural tendencies toward shame and a legal framework that potentially criminalizes medical errors. Support systems in Japan must go beyond standard mental health care to address these structural and legal barriers, ensuring true psychological safety to protect both the medical workforce and patient safety.

## Supplementary Information

Below is the link to the electronic supplementary material.

Supplementary Material 1.

## Data Availability

The datasets analyzed during the current study are not publicly available to protect the privacy of the participants. However, the data are available from the corresponding author on reasonable request, subject to approval by the relevant institutional review boards.

## Abbreviations

ASD: Absolute standardized differences
PSM: Propensity score matching
SVS: Second victim syndrome
STROBE: Strengthening the Reporting of Observational Studies in Epidemiology
