# Supplementary material for: The impact of medical errors on healthcare professionals: a cross-sectional propensity score-matched comparison of second victim syndrome between Japan and the United States
Source: Crit Care. 2026 Jul 31;30:408. doi: 10.1186/s13054-026-06212-5 (PMC13463400; doi:10.1186/s13054-026-06212-5)
Supplement: Supplementary file 1 [file 13054_2026_6212_MOESM1_ESM.docx]

The Impact of Medical Errors on Healthcare Professionals: A Propensity Score-Matched Comparison of Second Victim Syndrome between Japan and the United States

Comparison of Survey Instruments: Original (English) and Japanese Version

The survey questionnaire used in this study was adapted from the original survey developed by Kaur et al. (2012). The Japanese version was developed through a process of forward and backward translation. Some items were modified or simplified to fit the Japanese clinical context. The item-by-item comparison is presented below, in the order presented to the participants.

**Part 1: Demographics**

Q1. Profession

・English (Original): I am a(an): Attending physician / Fellow / Resident / Nurse / Pharmacist / Respiratory Therapist / Other

・Japanese (Used in this study): 職業を1つ選択してください

・Physician (Staff) / 医師（スタッフ）

・Physician (Resident) / 医師（レジデント）

・Nurse / 看護師

・Pharmacist / 薬剤師

・Clinical Engineer / 臨床工学技士

・Rehabilitation Therapist / リハビリ

・Other / その他

**Note on Adaptation**

Job categories were adjusted to reflect the Japanese healthcare system. For example, "Respiratory Therapist" was revised to "Clinical Engineer" (the position that typically manages ventilators in Japan), and the "Nurse Practitioner/Physician Assistant" categories were omitted as these roles did not exist in the same capacity in Japan at the time of the survey.

Q2. Specialty

・English (Original): My primary area of focus is: Internal medicine / Surgery / Anesthesiology / Pediatrics / Neurology / Emergency medicine / Other

・Japanese (Used in this study): 専門領域を１つ選択してください

・Emergency & Critical Care / 救急集中治療

・Anesthesiology / 麻酔

・Stroke / 脳卒中

・Internal Medicine / 内科

・Surgery / 外科

・Pediatrics / 小児

・Other / その他

Q3. Years of Experience

・English (Original): How many years of experience do you have in your current position?

・Japanese (Used in this study): 現在の職業の経験年数を１つ選択してください

・In training / 現在修練中

・0–5 years / ０－５年

・6–10 years / ６－１０年

・11+ years / １１年以上

・Other / その他

**Part 2: Scenarios** (Case Vignettes)

Participants were presented with three clinical scenarios. For each case, participants evaluated the likelihood of "Criticism from colleagues" and "Formal disciplinary action" across three outcomes (Minor, Moderate, and Fatal).

**Case 1: Procedural Error**

・Scenario (English): You are placing a central line in a patient. You are in a rush and skip the "timeout." The patient suffers a pneumothorax and requires a chest tube.

・Scenario (Japanese): 現在あなたは患者の中心静脈路を確保（またはその介助）をしています。手技の過程で、誤って動脈を損傷しました。

Outcomes Evaluated:

1. No complications (Minor): 合併症は起こらなかった場合
2. Extended hospital stay (Moderate): 緊急手術となり患者は回復したが、入院期間が1週間延長した場合
3. Patient death (Fatal): 患者が死亡した場合

Questions for each outcome:

・Q: Likelihood of Criticism from Colleagues

・English: What is the likelihood that you will be criticized by your colleagues?

・Japanese: 周りの医療スタッフから批判される可能性を1つ選択して下さい。

・Q: Likelihood of Formal Disciplinary Action

・English: What is the likelihood that you will receive formal disciplinary action?

・Japanese: 正式に懲戒処分を受ける可能性を1つ選択して下さい。

**Case 2: Diagnostic Error**

・Scenario (English Original): You or another credentialed critical care professional is evaluating a patient with severe chest pain with radiation to the back. Acute coronary syndrome is suspected and the patient is started on a heparin drip. A chest x-ray shows a widened mediastinum concerning for an aortic dissection. Another physician notes the chest x-ray, stops the heparin and sends the patient for repair of a confirmed aortic dissection.

・Scenario (Japanese): 現在あなたは胸痛を訴える患者を診察（または担当）しています。急性冠症候群を疑い、ヘパリンの点滴を開始しました。しかし別の医師が、胸部レントゲンの縦隔拡大の異常所見に気付き、ヘパリンをすぐ中止し、結局大動脈解離の治療が開始されました。

Outcomes Evaluated:

1. No harm (Minor): ヘパリン投与による影響はなく、患者が順調に治癒した場合
2. Extended hospital stay (Moderate): 貧血が進行しショックバイタルとなり、昇圧剤および輸血が必要になりました。その後回復しましたが入院期間が延長した場合
3. Patient death (Fatal): 貧血が進行しショックから心肺停止となり、患者が死亡した場合

Questions for each outcome: (Identical to Case 1)

**Case 3: Medication Error**

・Scenario (English Original): You or another credentialed critical care professional is working in the intensive care unit on a patient admitted with respiratory failure and pneumonia. A penicillin allergy is noted on admission and Ceftriaxone is ordered for him.

・Scenario (Japanese Version): あなたは肺炎による呼吸不全で入院した患者を診察（または担当）しています。この患者は入院時にペニシリンアレルギーの登録がされていましたが、あなたはペニシリンを投与してしまいました。

**Note on Modification**

In the original US survey, the scenario involved a potential cross-reactivity error (ordering ceftriaxone for a patient allergic to penicillin). In the Japanese version, this was simplified to a direct error (ordering penicillin) to ensure clarity regarding the contraindication.

Outcomes Evaluated:

1. No adverse effect (Minor): 副作用は特に起きなかった場合
2. Anaphylaxis recovered (Moderate): アナフィラキシーショックを起こしましたが、治療を行い完全に回復した場合
3. Death from anaphylaxis (Fatal): アナフィラキシーを起こし、死亡した場合

Questions for each outcome: (Identical to Case 1)

**Part 3: Post-Event Questions**

Q4. Emotional Responses

・English (Original): Following a severe adverse event, which of the following might you experience? (Select all that apply)

・Japanese (Used in this study): 医療事故が万が一重篤な転帰・死亡を引き起こした際に、次のうち想定される項目を全て選んで下さい。

・Items (Comparison):

・Anxiety (不安)

・Guilt (罪の意識)

・Reliving the event repeatedly (後から何度も思い出す)

・Fear of litigation (訴訟を恐れる)

・Depression (うつ)

・Loss of Sleep (眠れない)

・Loss of interest in daily activities (日常生活に無関心になる)

・Loss of concentration (集中力の低下)

・Fear of judgement from colleagues (周りの医療スタッフからの批判を恐れる)

・Anger at self (自分自身への怒り)

・Anger at others (他者への怒り)

・Professional self-doubt (仕事上の自信喪失)

・Defensiveness (自己防衛的になる)

・Loss of reputation (評判を落とす)

・Considered career change (転職を考える)

・Used alcohol or other substances (アルコールやその他の嗜好に走る)

・Shame (恥ずかしく思う)

・Thoughts of hurting oneself (自傷を考える)

・I did not experience any of these negative feelings (上記のような否定的な感情は経験しな

い)

・Item Regarding Past Involvement (Modified):

・English (Original): "Was never involved in a medical error that led to severe mobility or death"

・Japanese (Used in this study): "Was actually involved in a medical error leading to severe outcome or death" (実際に、重篤な転帰・死亡に至る医療事故に関わったことがある)

**Note on Modification**

The original survey inquired about past involvement using a negative statement ("Never involved"). The Japanese version rephrased this as a positive statement ("Actually involved") to assess the same experience.

Q5. Perceived Institutional Support

・Japanese (Used in this study): あなたの施設や上司は、そのような医療事故に対して協力的でしょうか？

・English Translation: Is your institution or supervisor supportive regarding such medical errors?

・Options: Yes (はい) / No (いいえ)

Q6. Behavioral Responses

・English (Original): Following such an event are you most likely to: (Select all that apply)

・Japanese (Used in this study): 重大な有害事象を起こした後、あなたが最も取る可能性が高い行動は次のうちどれですか？

・Options

・Talk extensively with colleagues about the event / 有害事象を広く周りの医療スタッフに話

す

・Talk extensively with friends or family about the event / 有害事象を広く友人や家族に話す

・Try to minimize the event and "just move on" / 有害事象をできるだけ最小限に留めようと

して、「ただ前に進む」

・Actively try to learn more about the issue / 積極的に問題点をより考えようとする

・Search for the "root cause" / 根本的な原因を探る

・Avoid the topic completely / 全く話題にしない

・Reject responsibility / 責任逃れをする

・Other / その他

Q7. Perceived Helpful Factors

・English (Original): What would be the most helpful to you following a severe adverse event? (Select all that apply)

・Japanese (Used in this study): 重大な有害事象を起こした後、次のうちどれが一番あなたにとって助けになりますか？

・Options

・Talking to colleagues / 周りの医療スタッフと話す

・Debriefing with the entire team / 関係したチーム全体で振り返る

・Talking to the patient or patient's family / 患者やその家族と話す

・Talking to a professional counselor / 専門的なカウンセラーと話す

・Talking to an attorney / 弁護士と話す

・Departmental Case Review / 所属科内での症例検討会

・Confidential Hospital Quality Review / 病院の医療の質と安全に関する検討会

・Other / その他
